# Energy Minimization Algorithm for Estimation of Clock Skew and Reception Window Selection in Wireless Networks

**DOI:** 10.3390/s21051768

**Published:** 2021-03-04

**Authors:** Michał Gorawski, Krzysztof Grochla, Rafał Marjasz, Artur Frankiewicz

**Affiliations:** 1 Institute of Theoretical and Applied Informatics, Polish Academy of Sciences, ul. Bałtycka 5, 44-100 Gliwice, Poland; mgorawski@iitis.pl (M.G.); rmarjasz@iitis.pl (R.M.); 2 AIUT Sp. z o.o., Wyczółkowskiego 113, 44-100 Gliwice, Poland; afrankiewicz@aiut.com

**Keywords:** LP WAN, LoRa, clock synchronization, time skew

## Abstract

The synchronization of time between devices is one of the more important and challenging problems in wireless networks. We discuss the problem of maximization of the probability of receiving a message from a device using a limited listening time window to minimize energy utilization. We propose a solution to two important problems in wireless networks of battery-powered devices: a method of establishing a connection with a device that has been disconnected from the system for a long time and developed unknown skew and also two approaches to follow-up clock synchronization using the confidence interval method. We start with the analysis of measurements of clock skew. The algorithms are evaluated using extensive simulations and we discuss the selection of parameters balancing between minimizing the energy utilization and maximizing the probability of reception of the message. We show that the selection of a time window of growing size requires less energy to receive a packet than using the same size of time window repeated multiple times. The shifting of reception windows can further decrease the energy cost if lower packet reception probability is acceptable. We also propose and evaluate an algorithm scaling the reception window size to the interval between the packet transmission.

## 1. Introduction

In wireless network systems, the synchronization of device clocks is an important factor, as it allows minimization of the energy use during communication by limiting the amount of time a radio interface operates in listening mode. It also allows synchronizing the data transmitted by different devices. In particular, industrial-based Internet of Things (IoT) installations, especially the ones that monitor complex systems or have strict energy consumption constraints, need an implementation of reliable clock synchronization mechanisms and methods of clock offset and clock skew estimation. The problem of clock skew is a well-documented phenomenon and is not new to scientists, as the clocks providing high accuracy are very expensive and low-cost devices need to operate with time sources having limited accuracy. Many implementations of distributed sensor systems require the nodes to work in a synchronized manner.

Battery-operated devices listening for radio messages transmitted by other devices devote a significant amount of energy to listening for incoming transmissions. Idle listening is a dominant source of energy consumption in many wireless communications standards [1,2]. Most existing protocols, such as the 802.11 power-saving modes (PSMs), attempt to reduce the time spent in idle listening by a sleep schedule. However, in sensor networks and low-power wide area networks (LP WANs), such as Long Range (LoRa) used in direct communication between battery-powered devices, the receiver knows the expected time of the transmission, but does not know the time skew of the transmitted clock. The drift and skew of the transmitter’s clock need to be estimated to select the listening time window that has a limited duration but assures a very low probability that the transmitter tries to send the message before or after the time when the receiver is listening.

The problem of clock synchronization in distributed systems has been heavily analyzed in the literature [3]. Especially in sensor networks, the collected information and measurements need to be merged to assemble a broader picture of the environment, which requires clock synchronization to perform data fusion functions. Clock synchronization functions have been built within many wireless protocols and are provided by cellular networks such as Long Term Evolution (LTE) [4,5]. Nevertheless, the growing popularity of low-power wide-area networks, such as LoRa, shows that there are still some use cases requiring clock synchronization and clock drift estimation not fully covered in the literature. Although the LoRaWAN protocol includes the function of clock synchronization with the gateways [6], the synchronization between battery-operated devices in LP WAN is still an open challenge.

In this paper we focus on sensor networks where smart devices collect measurements in various smart city systems, such as parking space monitoring, energy, water, gas consumption, etc., communicating using LP WAN. Each of these devices sends its measurements to a hub at a certain timestamp. With the inner skew of a quartz clock installed in these devices and other factors like temperature, humidity, etc., every device develops its own unique skew that has to be taken into consideration while collecting its data. Most of the smart devices operate using a battery source, so knowing a precise measurement’s time of arrival is crucial for energy-saving and battery longevity. Thus the problem of clock synchronization and skew approximation is challenging and important in such networks. In the IoT sensor systems, both the receiver and the transmitter have their clock shifted, especially the ones exposed to extreme weather conditions, and taking into consideration different skews of different devices increases the complexity of the problem.

We concentrate our research on two use cases:

(1) reception of the first packet from a newly deployed device, not synchronized for a longer period of time,
(2) continuous clock synchronization and optimization of the listening window’s size.

We assume that the receiver is a battery-operated device and tries to minimize the amount of time with a radio interface operating in listening mode. As has been shown e.g., in [7], the radio receiving mode constitutes a big chunk of energy utilization in wireless sensor nodes due to the much longer listening time than transmission time. The receiver can calculate the expected time of data transmission but does not know the clock skew of the transmitter. The transmitter sends a pack on a pre-defined interval (e.g., once per day) and has the clock synchronized during the production process. After some time (e.g., a few months) the devices have been installed and start the transmission using a radio interface.

We analyze the problem regarding how to (1) receive the first packet from the transmitter with a minimum amount of energy used and (2) select the listening time window for the consecutive packets to balance between the maximization of the probability of packet reception and the energy utilization. While the second problem is widely documented, the problem of finding a long inactive network node still has room for analysis. We propose the algorithms for listening window time selection and evaluate it using simulation. Our propositions solve an existing and important problem of reconnection of the device disconnected from the system for a longer period of time, and increase the efficiency of current methods implementing in the system in case of a follow-up clock synchronization and optimization of a listening window’s size. The proposed algorithm using listening windows of growing size or shifted in time allows decreasing the amount of energy used for listening by between 10 and 30%, compared to a commonly used repetition of the same window size.

The rest of the paper is organized as follows: Section 2 contains the literature review, Section 3 formulates the problem and describes the model, Section 4 shows the results of the performance evaluation of the proposed algorithm. We finish with a short conclusion.

## 2. Literature Review

The problems of clock offset and clock skew are well known and many research and solution propositions are already used in wireless sensor systems. There are many surveys and propositions of clock skew approximation, simulation, and clock resynchronization. Ref. [3] presents an extensive survey and analysis of clock synchronization protocols for wireless networks, along with a classification of such protocols, and [8] presents another extensive survey focusing on two representative protocols—Reference Broadcasting Synchronization (RBS) and Timing-synch Protocol for Sensor Networks (TSPN). One of the most obvious methods to achieve general synchronization of network devices is the use of GPS to synchronize devices with UTC. One of the propositions was presented in [9] and later optimized in [10] by the same authors. Although the accuracy of this method is quite high, even with energy consumption optimization GPS still requires periodic uptime, and the more accurate synchronization we need the more frequent it becomes. In addition, this requires the device to be equipped with a GPS module, which is not always possible.

Microprocessor systems usually use crystal oscillators as a time source. These are electronic devices that use the mechanical resonance of a piezoelectric material crystal to provide a constant frequency signal [11]. The crystal clocks have drift and do not run at the same rate as a reference clock, gradually desynchronizing from the other clocks. The frequency stability of the oscillator is subject to multiple factors affecting crystal oscillator frequency accuracy, such as temperature, aging, electric current level, and retrace (when power is removed from an oscillator and re-applied on it again, the frequency of this oscillator will stabilize at a slightly different value) [12]. Apart from the external factors such as temperature, there are quite a few research papers showing that the crystal oscillator drift has a normal (Gaussian) distribution [13,14,15]. In previous research, some authors proposed using the Bayesian approach or [16], machine learning, and Kalman filters [17] to predict the drift. Nevertheless, it gives only the answer on the expected time skew, not showing how to define the listening window to receive a message transmitted by a device with a desynchronized clock.

The authors of [18] present a solution to the clock skew problem in a sensor wireless network environment similar to our case. The proposed algorithm uses a non-standard approach where data measurements are synchronized by a per-hop method, as the data travel through the network to the sink node, which is different from the usual approach of clock synchronization. The fact that the system is being synchronized using data timestamps gives it higher flexibility and ability to cope with network topology changes. Ref. [19] presents a synchronization mechanism with an approximation of how temperature influences clock skew and gives insight into how the clock is influenced by temperature and how to deal with these important problems in systems implemented in an environment heavily influenced by temperature shifts. A new method for access control and mitigation of interfering noise in time synchronization environments is presented in [20]. Another paper that focuses on clock synchronization in a wireless network environment is [21]; here the authors propose an internal distributed clock synchronization solution using the group neighborhood average, where group averaging of offset and skew rate value are calculated instead of a conventional point-to-point averaging method. The authors of [22] focus on clock synchronization in the industrial Internet of Things (IIoT) with LoRa networks. Paper [23] proposes an algorithm for joint clock-skew and range estimation based on weighted least squares (WLS) using the ideas of 2-D frequency estimation. The authors of [23] analyze the performance of the algorithm they proposed in [24]—the energy-efficient time synchronization scheme based on the asynchronous source clock frequency recovery (SCFR). An interesting approach can be found in [25], where the authors deal with Mobile Underwater Sensor Networks and propose an red Adaptive Power Efficient Time Synchronization—APE-synch protocol that uses Kalman filters to assess the clock skew and reduces energy consumption for hard-to-get-to underwater devices.

The time synchronization in LP WAN is covered by some of the protocols, but with limited accuracy. In particular, the LoRaWAN Application Layer Clock synchronization Specification [26], authored by the LoRa Alliance, proposes an application layer messaging over LoRaWAN to synchronize the real-time clock of an end-device to the network’s clock, but only with the second accuracy. In the paper [27], the enhanced event correlation algorithm has been proposed to support message transmission and critical data sharing in a correct real-time manner, which allows synchronizing clocks between devices during continuous transmission, but does not solve the problem of reachability of a device disconnected for a longer period. The authors of [28] proposed a channel-hopping scheme based on time synchronization, which integrates cryptographic channel selection with the time notion for the current communication and increases the security of the transmission, but does not solve the problem of how the clocks are about to be synchronized.

### Clock Skew Measurements

We have analyzed the skew of the clocks of IoT devices across a larger device population by selecting a production batch of 8581 devices for the test. Each device was individually tested by the following automated test procedure:

1. Tested device generates a voltage pulse with duration of exactly 500,000 µs. Pulse duration is generated based on internal crystal oscillator.
2. External tester with base accuracy << 1 ppm is used to measure real pulse width.
3. Tests (1) and (2) are repeated 10 times and mean value is calculated for each device.
4. Average of expected clock skew over 1,000,000 s of operation is calculated.

**Figure 1:**
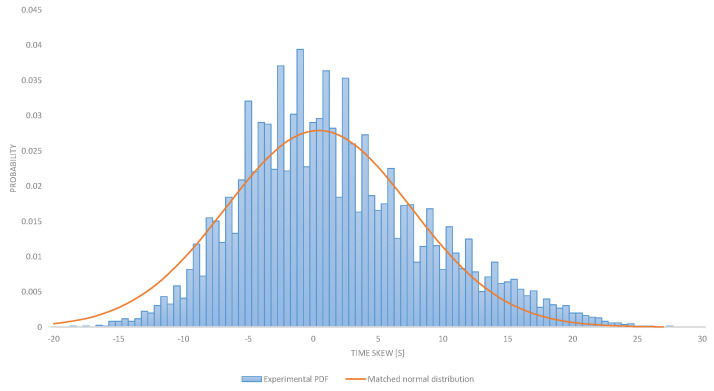
Clock desynchronization for stored devices, presented in parts per million.

All the devices were tested at room temperature after similar operation time. Possible measure inaccuracy like electrical pulse rises and fall times have been analyzed and were proven to cause less than 1 ppm inaccuracy. Figure 1 represents a histogram of population size divided by total device count also considered as probability (y axis) with given clock skew (x axis). The red line represents the fitted probability distribution function.

The observed distribution is very near to the normal distribution. These results are consistent with the phenomenon observed in the literature [13,14,15,29]. Based on this we believe that the normal distribution can be used to estimate the probability of the clock skew.

## 3. Proposed Solutions

We propose two algorithms to address the two problems identified in Section 1. First, we propose a time window selection scheme for an initial device connected to the network. Secondly, we propose an algorithm maintaining continuous communication with a device using the follow-up clock synchronization and optimization of the listening window’s size.

### 3.1. The Selection of a Listening Window to Establish a Connection with the Device

The scenario assumes a single synchronization of the transmitter device clock during its production. Afterwards, for a long period of time *X*, the device remains disconnected from the network and with unknown clock drift. After this period, we try to reconnect the device to the network by attempting to receive the signal transmitted from this device. We assume the quartz clock error as *Y*. The receiver sets the listening window according to the strategy defined and appropriately modified. We assume that the receiver should maximize the probability of reception of at least one packet within 3 consecutive transmission intervals (e.g., 3 days for a device transmitting a single packet per day). Traditionally, this is achieved by repetition of the same time window—e.g., if the receiver is expecting that the transmission occurs at 2 a.m. it listens e.g., from 1.59 a.m. to 2.01 a.m.

#### 3.1.1. Linear Increasing of Time Window

**Figure 2:**
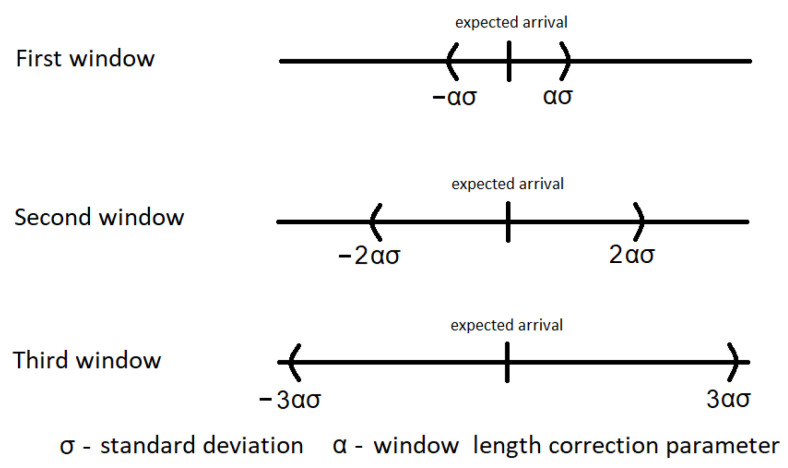
Linear increasing of time window.

We propose to listen for an incoming packet using a linearly growing time window. We set the first window to $x_{1}=2\cdot \left(\alpha \cdot \sigma \right)$, the second window to $x_{2}=2\cdot \left(2\cdot \alpha \cdot \sigma \right)$, and the third window to $x_{3}=3\cdot \left(2\cdot \alpha \cdot \sigma \right)$, where σ is the standard deviation of the normal distribution defining the clock-acceptable random values of the desynchronized device over appropriate period X[s], and α is a scaling factor. The center of the listening window is set as the point where we expect the data to be transmitted assuming a complete lack of clock desynchronization. The listening session is carried out as a three-window rule, the length and location in time are presented in Figure 2.

#### 3.1.2. Shifted Time Windows

**Figure 3:**
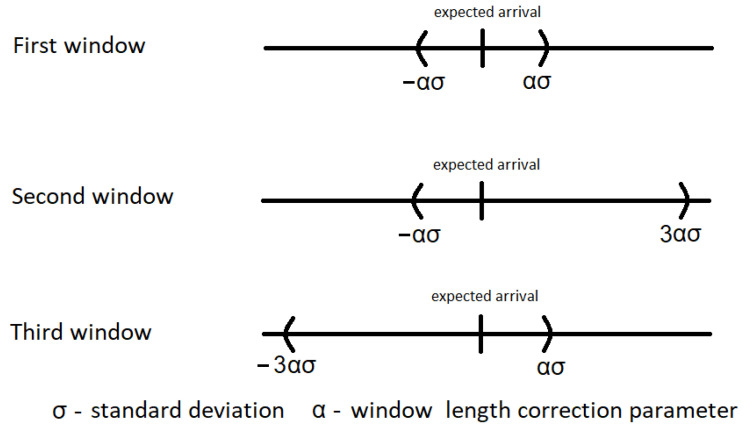
Shifted time windows.

The second proposed algorithm uses also three windows. The first one is centered around the expected reception time, the second one shifted up, and the third one is shifted down, as shown in Figure 3. The length of the windows, as previously, is scaled by the factor α. The duration of the first window is set to 2·α·σ, the second one to the interval <−3·α·σ,α·σ>, and the third one to <−α·σ,3·α·σ>. The shift scenario assumes the use of two asymmetric windows focused on the possibility of receiving packets from the two tails of the normal distribution, positive and negative in relation to the expected time of packet arrival.

### 3.2. Follow-Up Clock Synchronization and Optimization of Listening Window’s Size

The second problem we consider is how to select the time window for a consecutive transmission of packets, assuming we have received at least one packet from the transmitter. Assuming the interval between the last successful reception of the packet is known and the next packet is transmitted, the algorithm should define the starting and ending point of the listening window.

We analyze the following transmission scenario: the transmission takes place twice during each simulated day. Every single transmission takes place in one of two windows: between 0:00–3:00 and 3:00–6:00, and the exact moment of transmission in a given window is randomized according to a uniform distribution within the adopted limits. The receiver knows the expected time of arrival of each subsequent packet, while the actual time of its transmission by the transmitter is burdened with transmitter crystal skew.

The two reception windows solutions are presented as an emergency mechanism to assure the largest probability of packet reception. The transmissions are duplicated in each window to minimize the possibility of packet loss.

The solution was divided into two separate elements—the selection of the correct length of the listening window and the selection of a listening moment in time.

This time window management is based on a smart metering scenario, where the readouts of the meters need to be transmitted by a specific hour in the morning (in our example 6 am) and cover the reading of the water/energy/gas utilization at midnight. The proposed mechanism is generic and may be used for any time windows. The specific times are set as defaults in devices produced by AIUT company (www.aiut.com accessed on 29 December 2020), according to the real working system that was implemented in a few Polish cities for water and gas metering.

#### 3.2.1. Algorithms Selecting the Listening Moment

- An algorithm that synchronizes the receiver clock after each packet received (A1)
  When the packet is correctly received, the receiver clock is synchronized to its arrival time (i.e., the time of arrival becomes the “zero” time from which the time to the next expected packet arrival is counted).
- An algorithm of receiver time synchronization based on the statistics from the N most recent received transmissions (A2)
  The expected time of arrival of the next packet $t_{i+1}$ is determined on the basis of the receiver’s clock ${\tilde{t}}_{i}$ (the degree of desynchronization of the receiver’s clock towards the transmitter changes with the passage of time) and the calculated period $o_{i}$ after which the next transmission will occur: $t_{i+1}={\tilde{t}}_{i}+o_{i}$. The algorithm recalculates the interval *I*, the value of which is added to the expected time of arrival of the next packet ${\tilde{t}}_{i+1}=t_{i+1}+I={\tilde{t}}_{i}+o_{i}+I$. The interval is determined according to the formula:
  (1) $$\begin{array}{c} I=\hat{B}\cdot \left(t_{i+1}-{\tilde{t}}_{i}\right), \end{array}$$
  where
  (2) $$\begin{array}{c} \hat{B}=\frac{\sum_{i=1}^{N}\Delta B_{i}}{{\tilde{t}}_{N}-{\tilde{t}}_{1}}, \end{array}$$
  $\Delta B_{i}=tr_{i}-{\tilde{t}}_{i}$, and $tr_{i}$ is the time when *i*-th message actually arrives at the receiver.

#### 3.2.2. Algorithms Controlling the Selection of the Listening Window Length

- Window size adjustment algorithm based on the transmitter deviation (W1) The initial size of the window is determined based on empirical experience, and is then gradually increased or decreased according to the following relationships:
  - if the deviation of the next 10 (current + last 9) consecutive packet reception times (relative to the expected packet arrival time) is less than 30% of half of the current listening window length, then the window length is reduced by 30%;
  - if the deviation of the reception time of the last packet relative to its expected time of arrival is greater than 70% of half of the length of the current listening window, then the length of the window is increased by 30%;
  - if the packet is not received then the window length is increased by 30%.
- Window size adjustment algorithm based on the transmitter’s clock deviation with the continuously calculated probability of the window elongation (W2)
  The initial window size is determined by empirical experimentation, and then gradually increased or decreased according to the relationships presented in the algorithm (W1) with the following change:
  - if at the moment ${\tilde{t}}_{i}$ we denote the number of correctly received packets by cR, and the number of transmitted packets *R*, then with the probability of $\frac{cR}{R}$ we extend the length of the listening window. We also introduce a threshold $D_{min}$ of the calculated value $\frac{cR}{R}$, which is a minimum size of the window.
- Window size adjustment algorithm based on the statistics from the N most recent received transmissions (W3)
  The size *W* of the window is calculated from the formula $W=2\cdot A_{i}\cdot \left(t_{i+1}-{\tilde{t}}_{i}\right)\cdot \hat{B}$ taking into account the following dependencies:
  - if a packet is received then the next listening window length parameter is $A_{i+1}=A_{i}\cdot 0.9$, with condition $A_{i+1}\geq A_{min}$, where $A_{min}$ is empirically determined;
  - if no packet was received then the next listening window length parameter is $A_{i+1}=A_{i}\cdot 1.2$;
  - the N-element average is calculated from the Equation (2) presented in algorithm (A2), with the condition $\hat{B}\geq {\hat{B}}_{min}$, where the value ${\hat{B}}_{min}$ is established empirically.

**Figure 4:**
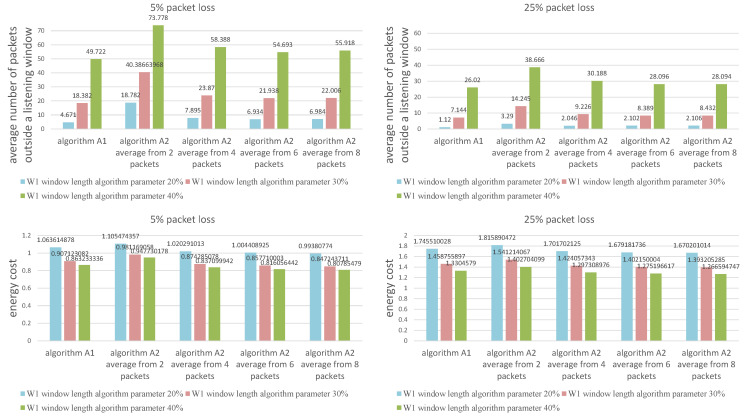
Comparison of window length change parameters.

We have chosen a 30 percent value (and proportionally 100% − 30% = 70% value) based on the experiments conducted via simulations. Figure 4 shows the different percentage values (namely 20 and 40% and their proportional counterparts) for two sizes of packet loss (5 and 25%). In both cases, we can observe that setting the value of the algorithm (W1) window length parameter to 30% keeps the desired balance between the average number of packets outside a listening window and the energy cost value (or the average window length value associated with energy consumption). Setting the value to 20% results in a significant increase in energy consumption, whilst the 40% value doubles or even triples the number of packages outside of a listening window to an unacceptable level.

## 4. Tests and Experiments

We evaluated the two proposed algorithms using a simulation, assuming the following parameters:

- initial interval before first transmission X=15,552,000[s] (180 days),
- standard deviation of time drift Y=0.000005 (which equals 5 parts per million (ppm)), consists of two parts: a constant drift randomly selected from the normal distribution, with a standard deviation of 2.5 ppm, and an additional skew also with a standard deviation of 2.5 ppm randomized for each interval.
- uniform packet loss model, with loss probability set to 5, 10, 20 and 30%.

The research was conducted on two separate simulation models, covering both problems discussed in the paper:

- Reconnection of the device disconnected from the system for a longer period of time.
- Follow-up clock synchronization and optimization of listening window’s size.

The research was conducted on two separate simulation models, covering both problems discussed in the paper:

- Reconnection of the device disconnected from the system was performed on simulation made in Microsoft Excel. We created two sheets, one with 140 thousand rows and the second with one million rows (each row represents one device), performing thorough research covering a multitude of independent time skew possibilities. Each row consists of time skew values for 3 consecutive transmission intervals calculated according to a normal distribution (see Section 3.2); qualification formulas to determine if the time burdened with transmitter crystal skew is inside a window range; energy consumption formulas to calculate overall cost for transmission reception.
- Follow-up clock synchronization and optimization of listening window’s size was performed on a simulation model made in the Objective Modular Network Testbed in C++ (OMNeT++), a modular, component-based C++ simulation library and framework. We programmed a complex behavior of a receiver module listening to the transmitter module packets sent accordingly to a transmission scenario in times burdened with transmitter crystal skew error.

### 4.1. Contacting the Device Disconnected from the Network for a Long Time Period

The first set of tests was executed to evaluate how the size of the window influences the probability of the packet reception. The repetition of the same reception window three times (denoted on the plot as the uniform scenario) is used as a baseline, where its duration is set to <−2·α·σ,2·α·σ> around the time of expected reception of the packet. We evaluated the influence of the scaling parameter α, set in the range from 0.3 to 1.4 in steps of 0.1. We assumed that 5% packets are lost due to radio transmission errors. Random moments of packet delivery to the listening device are calculated from the normal cumulative distribution for the specified expected arrival moment (after 180 for the first window and after 1 day for second and third) and standard deviation equal to 0.0005% of the period length (see Table 1). The energy cost is depicted as the average listening time in seconds, as the energy utilization is linearly proportional to the listening time. When a packet arrives during a listening window, the cost includes the time from the window start moment till the time of packet transmission end. If a packet is not received, the whole window length is added to the overall energy cost. The simulations were carried out on a sample of 140 thousand and a sample of one million random values determining the desynchronization of the device clock for both scenarios.

**Figure 5:**
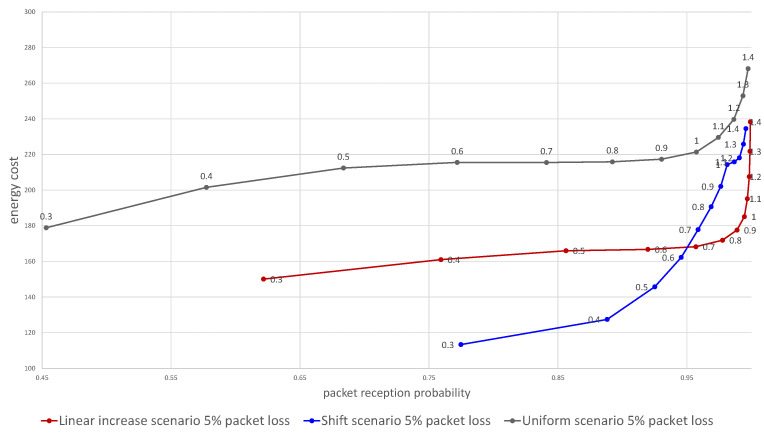
Dependence of the energy cost on the probability of packet delivery for the two proposed algorithms in comparison with using constant size time window. Numbers above data points denote α value.

The plot in Figure 5 shows the average energy utilization and packet reception probability for three tries (after three days). Each point represents an average of a set of 140 thousand simulations and shows for a given scaling parameter α the calculated reception probability and energy cost. By changing the α parameter the overall probability of getting at least one message can be adjusted, at the cost of the growing energy utilization. As the same α can result in different packet reception probabilities for the different algorithms, by putting it on a single plot we can compare the energy efficiency for a required packet reception probability or vice versa. The results of the simulation analysis presented in Figure 5 prove that both the shifted and the linearly increased time window allowed to achieve energy utilization lower by between 10 and 30 percent.

**Figure 6:**
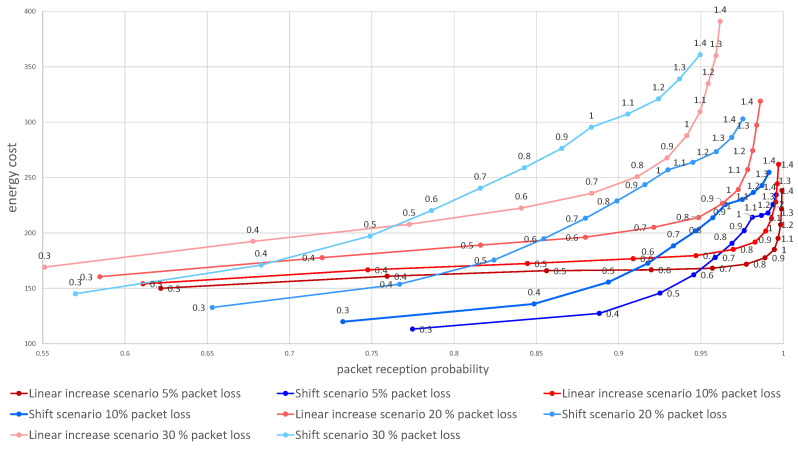
Dependence of the energy cost on the probability of packet delivery. Numbers above data points denote α value.

Next, we evaluated the influence of the packet loss ratio on the effectiveness of the two algorithms. Figure 6 visualizes the dependence of the energy cost on the probability of reception of at least one packet over the three tries for 5 to 30% packets lost due to wireless channel errors. The graph shows the overall advantage of the uniform linear increase of time window scenario (Section 3.1.1) over the shift scenario (Section 3.1.2) when lower overall reception probability is acceptable. However, when the packet loss ratio is low or if the lower probability of message reception is needed the shift algorithm outperforms the linear increase, providing lower energy utilization for any alpha below 0.5.

**Figure 7:**
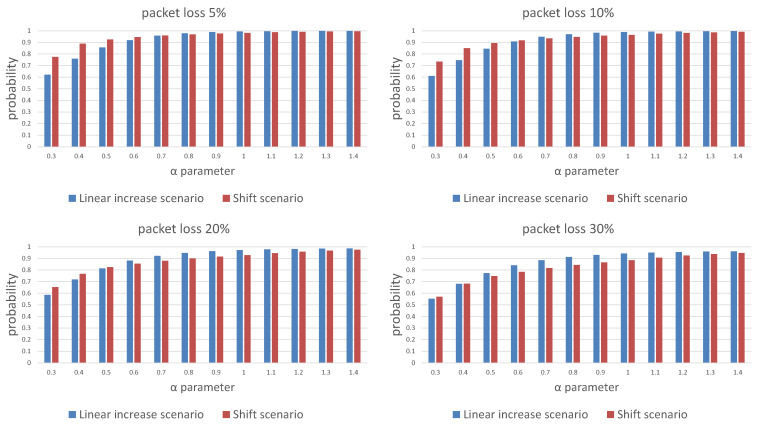
Comparison of the message reception probability over three trials for different packet loss probability for the linear window size increase and window shift algorithms.

**Figure 8:**
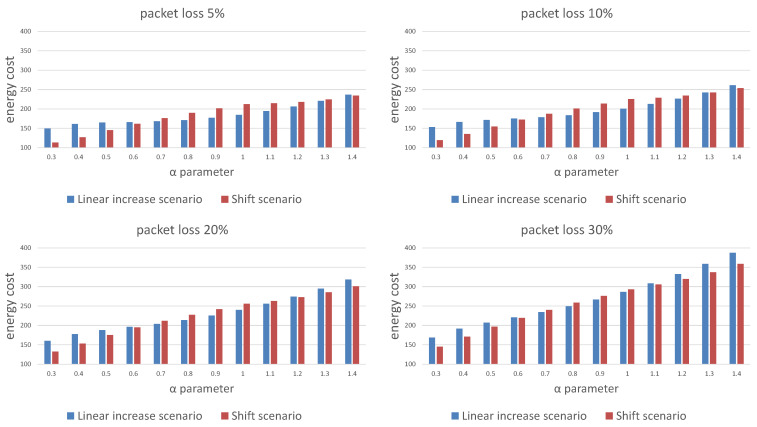
Comparison of the energy cost for different packet loss probability for the linear window size increase and window shift algorithms.

Figure 7 and Figure 8 show a comparison of the probabilities of receiving a package (Figure 7) and energy cost estimates (Figure 8) for both scenarios of window length selection for three consecutive listening windows. Our results show that any attempt to reduce the energy cost is associated with a decrease in the probability of receiving a packet. Depending on the required packet reception probability, α should be selected in the range [0.6,1]. An additional conclusion is a difference in the effectiveness of the strategy depending on the packet loss in the network. The linear increase of the window size scenario is less energy costly in networks with lower packet loss probability and also in a certain range of the α parameter values, the ends of which are visible on the charts and depend on the adopted packet loss.

### 4.2. Continuous Clock Synchronization and Optimization of Listening Window Size in Periodically Scheduled Transmissions

In the next step, we evaluated the combinations of the two proposed algorithms for estimation of the point in time at which the transmission occurs (algorithms A1 and A2 described in Section 3.2.1) and the three methods used to determine the listening window length (algorithms W1, W2, and W3 described in Section 3.2.2). The algorithm A2 is based on the average of the reception time of the last few packets, thus we have also evaluated how it performs using means $\hat{B}$ calculated from the statistics from 2, 4, 6, and 8 recently received packets. Each time, 1000 simulations of each combination were carried out, where a single simulation had an individual seed for the random number generator set out of 1000, which determined the times of packet transmission randomly selected in the windows 0:00–3:00 and 3:00–6:00 for each simulated day. Identical 1000 seed values were used for each of the combinations, ensuring the consistency of the compared results (the randomly selected transmission moments are the same for variable combinations of algorithms). The probability of packet loss assumed in the simulations changes between experiments in the discrete range {1%, 5%, 15%, 25%}.

**Figure 9:**
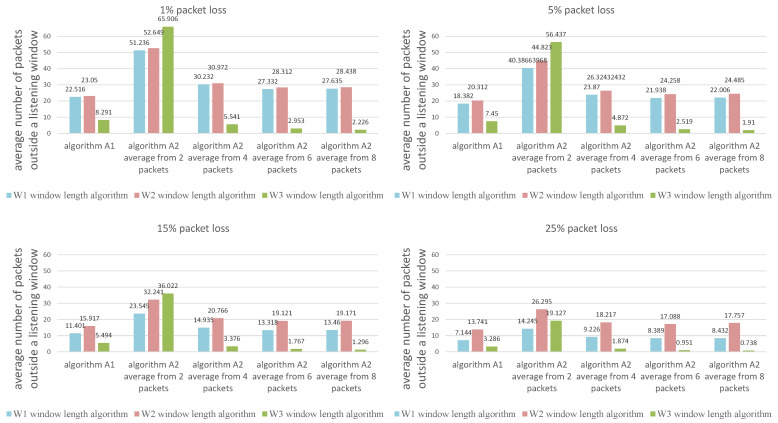
Comparison of algorithms in terms of the average number of packets outside the listening window.

**Figure 10:**
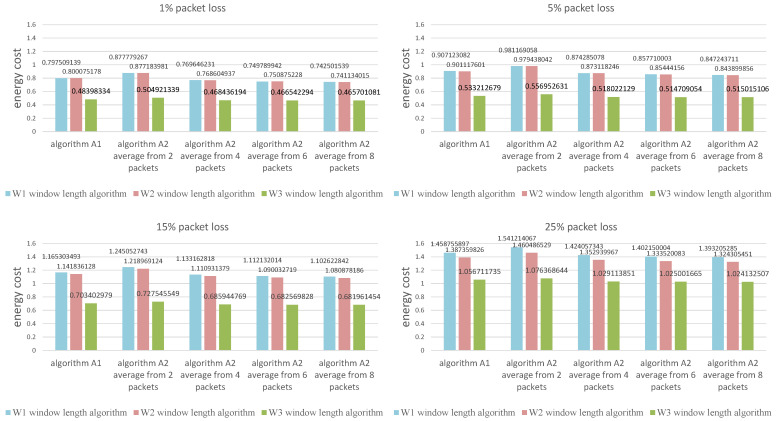
Comparison of algorith ms in terms of the average amount of energy used for listening during a single transmission.

**Figure 11:**
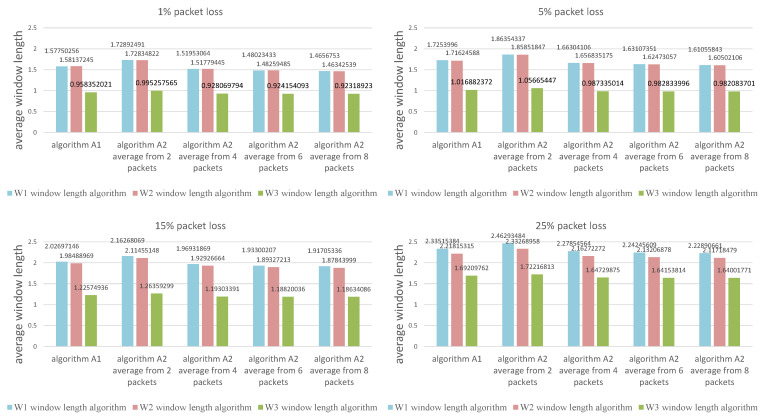
Comparison of algorithms in terms of the average length of the listening window.

**Figure 12:**
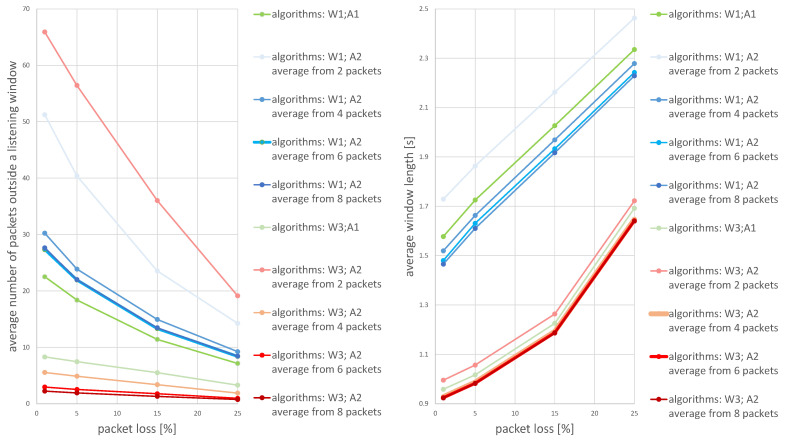
The degree of improvement brought by the algorithm (W3) in relation to the algorithm (W1) —algorithm (W2) is omitted in this statement because it did not bring a significant improvement.

The values presented in the figures are the average calculated from 1000 individual simulations, namely the average number of packets outside the listening window (Figure 9), the average amount of energy used for listening during a single transmission (Figure 10) and the average length of the listening window (Figure 11). Figure 12 shows the comparison between algorithms (W1) and (W3) combinations with (A1) and (A2) algorithms for a better view on the degree of improvement. The following parameter constant values were used in the algorithms for selecting the window length: $D_{min}=0.5$; $A_{min}=3.0$; ${\hat{B}}_{min}=0.0000035$.

Figure 12 provides insight into how all the evaluated methods are effective in terms of energy used for listening (the average window length) and the number of packets lost during simulation time. The combination of algorithms W3 (window width adjustment based on the average of the N most recent received transmissions) and A2 (receiver time synchronization based on the statistics from the N most recent received transmissions) outperforms all other methods. Generally, the higher the average number of time points used to calculate the average for algorithm A2 the better, but there is a very small difference between using 6 and 8 packets.

## 5. Discussion

Regarding the estimation of the clock skew to contact the device disconnected from the network for a long time period, a similar approach has been presented in the paper [29], in which the estimation of the clock skew distribution is also based on the normal distribution. However, instead of defining the window length, this paper just proposes a method to estimate the probability mass within a specific time frame and does not consider the shift or different sizes of the time window. Therefore it provides a more exact mathematical model to the calculation’s reference uniform scenario (repetition of the same reception window three times) method. Our proposition allows us to decrease the energy cost by up to 30% compared to such a simple solution, as was presented in Figure 5.

The continuous-time synchronization problem in most of the research papers is solved by messages updating the clocks in the network, e.g., in the flooding proposition presented in [30]. The algorithm proposed in this paper is more suitable for networks with rare communication (e.g., devices transmitting data once or twice per day), where clock synchronization would be energy demanding. A similar approach for a synchronization algorithm for a multihop network, which dynamically changes the window size to match clock drift, is presented in [31]. While the proposition is interesting, it assumes regular communication between nodes with additional synchronization communications exchanged between parent–child nodes and even more to obtain the global network synchronization. This is denoted as algorithm A1 on the plots. A recent paper by Shi et al. [29] proposed a novel maximum likelihood estimation (MLE) with an innovative implementation to minimize the clock skew estimation error caused by delay. The method presented in this paper allows selecting the listening window size to estimate the probability density of clock skew based on Gaussian delay. We further extend this concept by proposing how to synchronize the clocks during a continuous transmission, simplifying the decision threshold to decrease and increase the reception window size. We leave the possible merging of the two approaches for the proposed estimation method in [29], with our proposition of averaging the latest measurements for further study.

## 6. Conclusions

We have proposed a solution to two important problems in wireless networks of battery-powered devices: a method of establishing a connection with a device that has been disconnected from the system for a long time and developed unknown skew, and also two approaches to follow-up clock synchronization using the confidence interval method. The algorithms were evaluated using extensive simulations and we discussed the selection of parameters balancing between minimizing the energy utilization and maximizing the probability of reception of the message. We showed that the selection of a time window of growing size requires 10 to 30% less energy to receive a packet than using the same size of time window repeated multiple times. The shifting of reception windows can further decrease the energy cost if lower packet reception probability is acceptable. We also proposed and evaluated an algorithm scaling the reception window size to the interval between the packet transmission. The selection of the scaling parameter allows the energy cost to be balanced with the maximization of the number of packets received. Overall, the uniform linear increase in the time window scenario has the lowest energy cost for typical working conditions. For the expected probabilities of packet loss of 5 or 10%, assuming the value of the α=1 parameter allows the best balance of the energy cost with the maximization of the number of packets received.

We also discussed how to adjust the size of the reception window for continuous communication with a device. Our simulations show that using an average of six or eight last reception intervals provides a good estimation of both clock skew and drift and the proposed averaging method to scale the reception window size to the interval between packet transmission. We showed that such scaling allows achieving similar packet reception probability with a few times shorter listening times than a constant size reception window.

The recent popularity of LP WAN deployments raises new challenges for clock synchronization. Communication standards such as LoRa and MIoTy allow the low-cost battery-operated devices to operate for years, maintaining occasional communication with the network. As the bandwidth is very limited, the clock synchronization techniques based on a periodic exchange of packets are not effective. Some recent work has proposed flooding based synchronization [29], but the appropriate management of the listening window size and estimation of the clock skew, as proposed in [30] or this work, has strong potential to be applied in practice. In future, we plan to further extend the use of clock synchronization in communication between battery-powered LoRa devices and investigate the interoperability of this with the LoRaWAN standard, e.g., to minimize the size of the device’s listening window in LoRa classes A and B. Another area of future research is the application of more sophisticated prediction models, e.g., ARIMA for clock skew estimation.

## Figures and Tables

**Table 1:** Simulation parameters used in window length calculations.

	Days	X[s]	σ
First period	180	15,552,000	77.76
Second and third periods	1	86,400	0.432

## Data Availability

The data presented in this study are available on request from the corresponding author. The data are not publicly available due to consortium rules connected with project funding.
